# Inflammation

**Published:** 1907-12

**Authors:** 


**Inflammation.** By J. GEORGE ADAMI, M.A., M.D., F.R.C.S London: Macmillan & Co. 1907.

It was a happy thought which led Professor Adami to issue this reprint from his article on "Inflammation" in Allbutt's *System of Medicine.* Admirable as the original contribution was, the issue gains by the inclusion of the more recent work on the subject. Would that the book could find its way to the desk of every writer upon medical matters, and form a part of the outfit of all who carry out the practical applications of medicine and surgery!

"It cannot be too strongly emphasised," says Adami, "that a knowledge of the inflammatory process is the foundation of all pathology; and if our pathology is not to be a mere catalogue of names of morbid states, but an endeavour to bring into order and relationship the phenomena of disease, then the study of the inflammatory process is the natural starting-point for a right understanding of pathology and what it can teach us.

"It is deplorable that many surgeons enunciate a pathology at variance with that of the physicians; it is a matter for regret that general pathology and bacteriology are so rarely associated in one worker; but it must be acknowledged that no one man can hope to be equally familiar with the recent advances in neuropathology, hæmatology, immunity, infection, &c."

Hence the status of this book is at once defined. It marshals the facts already known; it states the deductions permissible, and points the way to further work; it tends to unify the terminology and the conception of inflammation; and as such, it should have the careful study of every student of medicine and surgery.

We are not going too far when we point out that in no other book is the subject so concisely and clearly set forth, and when we indicate that our lack of knowledge of its contents must stamp us as too little caring for the health of our patients; for Adami has not contented himself with the enunciation of principles, but has troubled to apply these to therapeutical details.

